# The Presentation of Eating Disorders in Saudi Arabia

**DOI:** 10.3389/fpsyg.2020.586706

**Published:** 2020-12-21

**Authors:** Aisha Jawed, Amy Harrison, Dagmara Dimitriou

**Affiliations:** ^1^ Sleep Education and Research Laboratory, UCL Institute of Education, London, United Kingdom; ^2^ Psychology and Human Development, UCL Institute of Education, London, United Kingdom

**Keywords:** eating disorders, Saudi Arabia, eating disorder examination questionnaire, eating disorder examination, validated measures

## Abstract

**Objective**: There is lack of information on the presentation of eating disorders (EDs) in Saudi Arabia using gold standard clinical tools. The present study aimed to provide data on the presentation of EDs in Saudi Arabia using clinically validated measures.

**Method**: Hundred and thirty-three individuals (33 male) with a mean age of 22 years (2.63) completed three measures: the Eating Disorder Examination (EDE), a semi-structured interview, the Eating Disorder Examination Questionnaire (EDE-Q), a self-report measure, and the Depression Anxiety and Stress Scale (DASS-21) to measure comorbid symptoms.

**Results**: Individuals in Saudi Arabia reported higher levels of restraint, eating concern and shape concern and a higher global score, but lower levels of weight concern on the EDE-Q compared to the EDE. Female participants reported a higher global score, alongside significantly higher scores on the restraint, shape concern and weight concern subscales than males. The most common ED subtype was other specific feeding or ED. Compared with Western community samples, symptom severity in this purposive sample obtained from community settings was significantly higher in this sample.

**Discussion**: Individuals with eating, weight and shape concerns in Saudi Arabia may feel more comfortable expressing their symptoms on a self-report tool compared with a face to face interview. However, it is possible that a self-report measure may over-estimate the severity of symptoms. The data suggest that clinicians in Saudi Arabia should regularly screen for EDs in all genders. It is also important to note that ED symptoms are a cause for concern in young people in Saudi Arabia.

## Introduction

A growing number of studies indicate a gradual increase in disordered eating behaviors in Western societies and some non-Western countries, with similar symptoms observed across these countries (Gunewardene et al., 2001; Huon et al., 2002). However, research on the clinical presentation of eating disorders (EDs) and morbidity, and mortality data (e.g., Herpertz-Dahlmann, 2009; Attia, 2010) remains largely focused on Western populations, namely the United States, United Kingdom, Australia and Western European countries (Scharff et al., 2019; Vanderlinden et al., 2020). What is of concern regarding the lack of research from non-Western settings is that many countries have gone through sociocultural changes and this process of acculturation has been shown to impact psychological wellbeing and eating behaviors (Thomas, 2013). Further, individuals in non-Western cultures such as those in the Middle East might be at increased risk for aberrant eating behaviors due to their emotional and religious connection to food (e.g., meaning of food and emotional attachment/importance), which may place non-Western cultures at an increased risk of developing EDs. Hence, it is of interest to explore the presentation of EDs in non-Western cultures, such as Saudi Arabia, an under-researched culture which hosts one of the youngest populations in the world.

To date, minimal data on the presentation of EDs in Saudi Arabia is available (Taha et al., 2018) with only a small handful of published studies which have used inventories such as the Eating Attitude Test (EAT-26; Garner et al., 1982; Alhazmi and Al Johani, 2019), the Sick-Control-One-stone-Fat-Food (SCOFF; Morgan et al., 1999; Aoun et al., 2015) or the Eating Disorders Inventory (EDI; Al-Subaie et al., 1996; Garner, 2004) to measure symptoms. In the most recent study conducted by Alhazmi and Al Johani (2019), 342 health specialties university students from a university in Saudi Arabia provided responses on the EAT-26. The authors identified 28.7% of respondents were at high risk of developing an ED. Risk was higher for females compared to males and positively associated with higher body mass index (BMI), age and grade point average. This indicates concerning levels of clinically significant ED symptoms among young people in Saudi Arabia. Although these findings are relevant, no previous study in Saudi Arabia has administered gold standard (Guest, 2000) symptom-reporting/diagnostic tools like the Eating Disorder Examination (EDE; Bohn and Fairburn, 2008) or the Eating Disorder Examination Questionnaire (EDE-Q; Fairburn and Beglin, 1994), which may limit potential comparisons with data from other countries where these measures are commonly used. Further, one advantage of using these tools over the other measures previously employed are that possible diagnoses can be offered and the nature of the symptoms can be clearly ascertained, such as the relative presence of different ED symptoms subtypes.

Therefore, this descriptive study aimed to administer the EDE and the EDE-Q to males and females to provide a clear description of the presentation of EDs in Saudi Arabia. A further objective was to explore the consistency of the interview-based inventory (EDE) and the self-report inventory (EDE-Q) in this population and to offer comparisons with data from Western populations. It was hypothesized that symptoms, measured using the EDE and EDE-Q would be higher among female compared to male participants; that the EDE and EDE-Q would identify similar symptoms and symptom severity and that the severity of symptoms, measured using the EDE and EDE-Q global scores in this Saudi Arabian sample would be similar to those reported in Western samples.

## Materials And Methods

### Design

This study used a between-participants design and involved a volunteer, purposive sample recruited from community settings.

### Participants

One hundred and thirty-three individuals (33 male) who were recruited *via* advertisements in hospitals, universities and social media platforms (Facebook, Instagram and Twitter) in Saudi Arabia participated in this study. The advertisements were placed in public places in a general hospital and university settings (not named to increase the anonymity of the sample) where we thought we might be likely to obtain a volunteer sample of individuals with possible EDs. Inclusion criteria were males and females who self-identified as having concerns about eating, shape, and weight, were sufficiently fluent in English to be able to respond to the measures, and aged between 18 and 35. Exclusion criteria were a history of brain trauma or the presence of a learning disability. We did not have any inclusion/exclusion criteria related to a prior diagnosis of EDs. This study is part of a larger project, which has involved collecting data from neuropsychological assessments validated in the English language, hence we were restricted to collecting data in English. Table 1 presents demographic characteristics for the sample.

**Table 1:** Demographics and Comorbidity data of full sample.

	Full sample	Gender		*t*(*df*)^a^/*χ* ^2^ ^b^	*p*	*d*
		Females	Males			
	(*N* = 133)	(*N* = 100)	(*N* = 33)			
Age (years)	22.17 (2.63)	21.77 (2.52)	23.38 (2.61)	−3.17 (131)^a^ [0.60/2.62]	0.002	0.06
BMI (kg/m^2^)	18.81 (1.02)	18.68 (0.98) Range: 15.99–20.89	19.23 (1.04) Range: 17.53–21.91	−2.74 (131)^a^ [0.15/0.95]	0.007	0.06
Education level				18.84 (2)^b^	<0.001	0.81
High school	4 (3.0)	0 (0.0)	4 (12.1)			
University	112 (84.2)	91 (91.0)	21 (63.6)			
Graduate	17 (12.8)	9 (9.0)	8 (24.2)			
School						
Employment				0.74 (1)^b^	0.390	0.15
Unemployed	115 (86.5)	85 (85.0)	30 (90.9)			
Employed	18 (13.5)	15 (15.0)	3 (9.1)			
Income (SAR)				10.12 (3)^b^	0.018	0.57
0	115 (86.5)	85 (85.0)	30 (90.9)			
1–100	2 (1.5)	2 (2.0)	0 (0.0)			
101–200	12 (9)	12 (12.0)	0 (0.0)			
201–300	4 (3)	1 (1.0)	3 (9.1)			
Children				2.88 (1)^b^	0.090	0.30
No	130 (97.7)	99 (99.0)	31 (93.9)			
Yes	3 (2.3)	1 (1.0)	2 (6.1)			
Marital status				2.44 (2)^b^	0.295	0.27
Single	126 (94.7)	96 (96.0)	30 (90.0)			
Married	6 (4.5)	3 (3.0)	3 (9.1)			
Divorced	1 (0.8)	1 (1.0)	0 (0.0)			
DASS						
Depression	15.82 (3.70)	17.54 (2.05)	10.61 (2.47)	16.00 (131)^a^ [6.07/7.79]	<0.001	3.21
Anxiety	13.70 (2.11)	13.58 (1.93)	14.06 (2.57)	−0.99 (44.55)^a^	0.330	0.02
Stress	15.93 (2.27)	15.12 (1.59)	18.39 (2.26)	[−0.46/1.22]−9.17 (131)^a^	<0.001	1.84
Total	45.45 (3.65)	46.24 (3.23)	43.06 (3.86)	[2.56/3.98] 4.67 (131)^a^ [1.83/4.53]	<0.001	0.94

Test statistics.

a Independent sample *t*-test comparing males and females.

b Chi square comparing males and females.

DASS, depression, anxiety and stress scale (Lovibond and Lovibond, 1995); SAR, Saudi Riyal; for information, 1 SAR = US$0.27. Applying the Bonferroni correction, significance was assumed based on a corrected alpha of 0.005.

### Measures

The *EDE* (Bohn and Fairburn, 2008): provides a measure of the type and severity of ED features. The tool comprises of four subscales: Restraint, Eating Concern, Shape Concern, and Weight Concern and a global score, computed as a mean of the subscales. The measure also assesseses specific behavioral symptoms, such as the frequency of binge eating, self-induced vomiting, laxative misuse, diuretic misuse, and excessive exercise. Items that assess the cognitive symptoms of EDs are based on a 28-day time frame and are scored on a seven-point Likert scale ranging from 0 to 6, with higher scores indicating more severe pathology. Participants provide frequencies when responding to the behavioral symptom items. In their review of psychometric studies of the EDE, Berg et al. (2012) found good test re-test reliability of 0.70–0.97 and inter-rater reliability of at least 0.90. They also report good internal consistency coefficients for the subscales ranging from 0.58 to 0.78 for the Restraint subscale, 0.44–0.78 for the Eating Concern subscale, 0.68–0.85 for the Shape Concern subscale, and 0.51–0.76 for the Weight Concern subscale. In this study there was a Cronbach’s alpha of 0.61 for the Restraint subscale, 0.59 for the Eating Concern subscale, 0.72 for the Shape Concern subscale and 0.57 for the Weight Concern subscale. (Fairburn et al., 1993)

The *EDE-Q* (Fairburn and Beglin, 1994): is a 28-item self-report questionnaire developed after the EDE, which measures psychopathological and behavioral indicators of disordered eating. Like the EDE, the EDE-Q has four subscales: Restraint, Eating Concern, Weight Concern, and Shape Concern, and the mean of these subscales provides the global score. Subscale and global scores range from 0 to 6, with higher values indicating greater symptoms. In Berg et al. (2012) review of 12 psychometric studies, it was shown to detect large sized differences between symptomatic and low/asymptomatic groups, offers acceptable internal consistency, with alphas ranging from 0.70 to 0.93, showed evidence of temporal stability of symptoms when measured over time (correlations ranging from 0.42 to 0.69 depending on symptoms assessed), offers good test re-test validity (test-retest correlations ranging from 0.66 to 0.94 for scores on the four subscales and from 0.51 to 0.92 for the behavior frequency items) and offers strong inter-rater reliability, particularly for the behavior frequency items, with inter-rater reliability coefficients ranging from 0.71 to 0.94 across the four subscales. In this study there was a Cronbach’s alpha of 0.63 for the Restraint subscale, 0.62 for the Eating Concern subscale, 0.69 for the Shape Concern subscale and 0.59 for the Weight Concern subscale.

The *Depression, Anxiety, and Stress-Scale* (*DASS-21*; Lovibond and Lovibond, 1995): is a 21-item measure with seven items in each of the three subscales: depression, anxiety and stress and the final score for each subscale is calculated and then it is multiplied by 2. This was included to measure common comorbidities within the sample. Antony et al. (1998) confirmed the presence of the three subscales in their confirmatory factor analysis and found good internal consistency with Cronbach’s alphas of 0.94 for depression, 0.87 for anxiety and 0.91 for stress. In this study, there was a Cronbach’s alpha of 0.91 for depression, 0.81 for anxiety and 0.85 for stress.

Participants were asked to provide demographic information on their age, education level, employment, income, marital status and number of children.

### Procedure

This study was approved by the Institute of Education UCL Research Ethics Committee Ref Z6364106/2018/07/44. All participants provided written informed consent prior to their inclusion in the study. The measures were administered individually, and in person. Participants first completed the EDE-Q, followed by the DASS-21 and then the EDE interview was then administered. The data were collected between June 2018 and October 2018 in Jeddah, Saudi Arabia.

### Statistical Analyses

Data were analyzed using SPSS software version 22. Normality tests including the Kolmogorov-Smirnov test and Skewness and Kurtosis values, and a review of boxplots and histograms suggested that the data were normally distributed within groups and therefore parametric tests were selected. Independent sample *t*-tests/paired-tests and Chi square tests were run. Comparisons with published data were conducted using a one sample *t*-test. As multiple tests of group differences were conducted for each analysis, the Bonferroni correction (0.05/K) was used to reduce Type 1 error. Effect size estimations were calculated based on Cohen’s *D* (Cohen, 1992) with 0.2 relating to a small effect, 0.5 relating to a medium effect, and 0.8 relating to a large effect. The diagnostic algorithm developed by Fairburn et al. (2018) for generating DSM 5 (American Psychiatric Association, 2013) and described here https://www.credo-oxford.com/7.2.html was used to generate the diagnostic subgroups.

## Results

Table 1 provides demographics and comorbidity data for the full sample and breaks these data down by gender. Males were significantly older than females, albeit with a small effect size. Males were more likely to have graduate school level education whereas females were more likely to have completed undergraduate studies. Based on the DASS-21, males and females reported similar levels of anxiety and females reported more depression than males, with a large effect size. Females were also less stressed than males, with a large effect size.

### Comparisons Between the Eating Disorder Examination and Eating Disorder Examination Questionnaire

Table 2 provides data from the EDE and EDE-Q for the entire sample and broken down by gender.

**Table 2:** Eating disorder measures for the full sample and by gender.

	Full sample	Gender		*t*(*df*)	*p*	*d*
		Females	Males			
	(*N* = 133)	(*N* = 100)	(*N* = 33)			
	*M*(*SD*)	*M*(*SD*)	*M*(*SD*)			
EDE-Q						
Restraint	4.62 (0.56)	4.77 (0.51)	4.15 (0.39)	6.43 (131)	≤0.001	1.29
Shape concern	5.41 (1.04)	4.84 (0.34)	4.44 (0.24)	6.29 (131)	≤0.001	1.26
Eating concern	3.54 (0.81)	3.25 (0.68)	4.2 (0.46)	−11.21	≤0.001	1.50
Weight concern	3.89 (0.58)	4.06 (0.56)	3.39 (0.32)	(80.48)	≤0.001	1.31
Global score	4.36 (0.39)	4.23 (0.33)	4.05 (0.23)	8.46 (95.38)	≤0.009	0.58
Behavioral Iitems (mean frequency, SD)						
Objective overeating	11.42 (2.32)	11.75 (2.52)	10.42 (1.06)	4.23 (123.66)	≤0.001	0.59
Loss of control	9.84 (2.16)	10.18 (2.24)	8.82 (1.56)	3.25 (131)	0.001	0.65
Objective bulimic episodes	7.09 (1.55)	6.84 (1.52)	7.85 (1.42)	−3.37 (131)	0.001	0.68
Vomiting	7.87 (2.65)	9.01 (1.93)	4.42 (1.09)	12.99 (131)	≤0.001	2.60
Laxative use	8.44 (2.26)	9.13 (1.86)	6.36 (2.12)	7.15 (131)	≤0.001	1.44
Excessive exercise	12.13 (3.71)	10.57 (2.51)	16.85 (2.50)	−12.47 (131)	≤0.001	2.50

Test statistics: independent sample *t*-tests comparing males and females. Applying the Bonferroni correction, significance was assumed based on a corrected alpha of (≤0.01) for each tool. EDE-Q, eating disorder examination questionnaire; EDE, eating disorder examination (Fairburn and Beglin, 1994). For behavioral items, data refer to the mean frequency in the previous 28 days period.

Comparisons of scores obtained from these two tools for the full sample showed significantly higher scores with large effect sizes on the restraint subscale of the EDE-Q [*t*(132) = 10.30, *p* < 0.001, *d* = 1.24]; shape concern scores [*t*(132) = 21.19, *p* < 0.001, *d* = 2.57] and global score [*t*(132) = 11.55, *p* < 0.001, *d* = 1.49] than obtained by the EDE. There was no difference in eating concern scores [*t*(132) = −0.34, *p* = 0.731, *d* = 0.04] between the two tools. Significantly lower scores were observed on the weight concern subscale on the EDE-Q [*t*(132) = −7.35, *p* < 0.001, *d* = 0.74] compared to the EDE.

### Possible Gender Differences in Symptoms Identified by the Eating Disorder Examination and Reported on the Eating Disorder Examination Questionnaire

On the EDE-Q (Table 2), females reported higher levels of restraint, shape concern, weight concern, and a higher global score, alongside lower levels of eating concern than males, with medium to large effect sizes. Males reported more objective bulimic episodes on the EDE-Q than females, with a medium effect size.

On the EDE, there were no gender differences in the restraint, shape concern or eating concern subscales, but females reported significantly higher levels of weight concern than males, with a large effect size and a significantly higher global score than males, with a medium effect size. There were no gender differences in objective bulimic episodes on the EDE.

### Eating Disorder Diagnostic Subtypes Based on the Eating Disorder Examination

Table 3 provides descriptive data on the percentage of ED subtypes for the whole sample and by gender. A Chi square test revealed that there were no gender differences in ED subtypes; *χ* ^2^(4) = 1.56, *p* = 0.820 (Fisher’s Exact Test = 1.78, *p* = 0.794; due to small cell count). The most common ED diagnosis was other specific feeding or ED (OSFED).

**Table 3:** Eating disorder diagnostic subtypes identified in the sample.

Subtypes	Full sample	Gender	
		Females	Males
	(*N* = 133)	(*N* = 100)	(*N* = 33)
	*N* (%)	*N* (%)	*N* (%)
AN-R	9 (6.8)	7 (7.0)	2 (6.1)
AN-BP	15 (11.3)	11 (11.0)	4 (12.1)
BN	13 (9.8)	8 (8.0)	5 (15.2)
BED	25 (18.8)	19 (19.0)	6 (18.2)
OFSED	71 (53.4)	55 (55.0)	16 (48.5)

AN-R, anorexia-nervosa restricting subtype; AN-BP, anorexia nervosa binge-purge subtype; BN, bulimia nervosa; BED, binge eating disorder and OSFED, other specified feeding or eating disorder.

### Comparison of Sample Data With Published Community Norms for the Eating Disorder Examination Questionnaire

Table 4 provides a comparison of the means for the EDE-Q for the current sample and two previous large community based studies incorporating roughly similar samples (females: Mond et al., 2006; males: Lavender et al., 2010). The participants in our sample reported significantly higher scores on all subscales with large effect sizes than those reported in these studies.

**Table 4:** Comparison of sample data with community norms reported for females by Mond et al. (2006) and for males by Lavender et al. (2010) for the eating disorder examination questionnaire.

	Mond et al., 2006	Present study	*t*(*df*)	*p* value	*d*
EDE-Q	Female sample (*N* = 5,255) *M* (SD)	Female sample (*N* = 100) *M* (SD)			
Restraint	1.30 (1.40)	4.77 (0.51)	67.51 (99)	<0.001	6.81
Shape concern	0.76 (1.06)	4.84 (0.34)	121.00 (99)	<0.001	12.22
Eating concern	1.79 (1.51)	3.25 (0.68)	21.62 (99)	<0.001	2.18
Weight concern	2.23 (1.65)	4.06 (0.56)	32.87 (99)	<0.001	3.32
Global score	1.52 (1.25)	4.23 (0.33)	82.72 (99)	<0.001	8.35
	Lavender et al., 2010	Present study	*t*(*df*)	*p* value	*d*
EDE-Q	Male sample (*N* = 404) *M* (*SD*)	Male sample (*N* = 33) *M* (*SD*)			
Restraint	1.04 (1.19)	4.15 (0.39)	45.96 (32)	≤0.001	2.70
Shape concern	1.59 (1.38)	4.44 (0.24)	68.23 (32)	≤0.001	2.14
Eating concern	0.43 (0.77)	4.42 (0.46)	49.85 (32)	≤001	5.31
Weight concern	1.29 (1.27)	3.39 (0.32)	37.22 (32)	≤001	1.71
Global score	1.09 (1.00)	4.77 (0.23)	91.41 (32)	<0.001	3.82

EDE-Q, eating disorder examination questionnaire (Fairburn and Beglin, 1994). Applying the Bonferroni correction, significance was assumed based on a corrected alpha of ≤0.01.

## Discussion

This study explored the presentation of EDs in Saudi Arabia, which for the first time, involved administering the EDE and EDE-Q to a volunteer sample of individuals with concerns about their eating, weight, and shape to better understand the nature and severity of EDs in this setting. The findings revealed that individuals in Saudi Arabia reported higher levels of restraint, eating and shape concern, and global scores, but lower levels of weight concern on the EDE-Q compared to the EDE. This suggests that in this setting, clinicians should take care when administering the EDE-Q alone, without a clinical interview, because these different assessment tools may yield different information.

On the EDE-Q, female participants reported a higher global score, alongside significantly higher scores on the restraint, shape concern and weight concern subscales than males, suggesting that females in Saudi Arabia may experience overall more severe symptoms than males. This finding also provides initial evidence that the two measures may be able to detect possible gender differences in ED symptoms in this population. One caveat to highlight here is that our study may have been underpowered given that 100 women and 33 men opted to take part and more work will be needed to further investigate these findings with larger sample sizes.

Comparisons between data from the EDE-Q with gender-matched community samples (Mond et al., 2006; Lavender et al., 2010) revealed that participants in the present sample reported significantly higher scores across all subscales, suggesting that the severity of ED symptoms in the community in Saudi Arabia could be higher than in similar Western samples. However, an important caveat to make with regards to this comparison is that our sample over-represents those with concerns about eating weight and shape, given that these were inclusion criteria for the study and the sampling was purposive in this sense. We also directly targeted places such as hospital settings, universities and online platforms where we thought we would encounter individuals with specific concerns about their eating, shape and weight which may perhaps have biased the sample towards a more severe symptom group, whereas Lavender et al. (2010), for example, sampled male undergraduate students and had a wider spectrum of symptomatic (and indeed asymptomatic) presentations. Nonetheless, our findings add to the growing literature on the presentation of ED symptoms in non-Western populations (Pike and Dunne, 2015) and as they indicate the presence of significant ED symptoms in Saudi Arabia, these data suggest that clinicians should regularly screen for EDs.

The finding, which most cases identified using the EDE were categorized as OSFED, is a similarity between this sample and those from Western populations (Machado et al., 2013). Further, 19% of the female sample and 18.2% of the male sample were identified as having Binge Eating Disorder (BED) which also mirrors findings that the gender ratio for this form of ED is roughly equal (Striegel-Moore et al., 2009).

These data have important implications for researchers interested in using the EDE or EDE-Q diagnostically. The results suggest that researchers interested in estimating the prevalence of EDs in this population might overestimate symptoms if using the EDE-Q relative to the EDE. However, it is also important to consider that the questionnaire version of the tool may be more amenable to this population who are more able to reflect on and disclose the nature of their symptoms in a self-report format and may be less open when engaging in a semi-structured interview. This may reflect a degree of stigma in relation to ED symptoms and future research should consider the acceptability of the tool for participants when considering how to collect information on ED symptom prevalence in larger samples in Saudi Arabia.

In conclusion, the present study provides the first data on ED symptoms using the EDE and EDE-Q in Saudi Arabia. There is a clear need to support clinicians to screen for EDs and to develop services to treat those with these disorders in this context.

## Data Availability Statement

The datasets presented in this article are not readily available because when the data were collected participants did not give permission for the data to be uploaded or shared to third parties, so we would need to ask participants’ permission first before sharing any data requests to access the datasets should be directed to DD: d.dimitriou@ucl.ac.uk.

## Ethics Statement

The studies involving human participants were reviewed and approved by UCL, Institute of Education (Z6364106/2018/07/44). The patients/participants provided their written informed consent to participate in this study.

## Author Contributions

All authors contributed to the development of this paper. AJ collected all the data. DD and AH contributed equally in the results section and overall write up of this manuscript. All authors contributed to the article and approved the submitted version.

### Conflict of Interest

The authors declare that the research was conducted in the absence of any commercial or financial relationships that could be construed as a potential conflict of interest.
